# POOE: predicting oomycete effectors based on a pre-trained large protein language model

**DOI:** 10.1128/msystems.01004-23

**Published:** 2023-12-11

**Authors:** Miao Zhao, Chenping Lei, Kewei Zhou, Yan Huang, Chen Fu, Shiping Yang, Ziding Zhang

**Affiliations:** 1 State Key Laboratory of Animal Biotech Breeding, College of Biological Sciences, China Agricultural University, Beijing, China; 2 School of Chemistry and Biological Engineering, University of Science and Technology Beijing, Beijing, China; 3 State Key Laboratory of Plant Environmental Resilience, College of Biological Sciences, China Agricultural University, Beijing, China; Drexel University, Philadelphia, Pennsylvania, USA

**Keywords:** effectors, oomycetes, prediction, bioinformatics, machine learning, protein language model

## Abstract

Oomycetes are fungus-like eukaryotic microorganisms which can cause catastrophic diseases in many plants. Successful infection of oomycetes depends highly on their effector proteins that are secreted into plant cells to subvert plant immunity. Thus, systematic identification of effectors from the oomycete proteomes remains an initial but crucial step in understanding plant–pathogen relationships. However, the number of experimentally identified oomycete effectors is still limited. Currently, only a few bioinformatics predictors exist to detect potential effectors, and their prediction performance needs to be improved. Here, we used the sequence embeddings from a pre-trained large protein language model (ProtTrans) as input and developed a support vector machine-based method called POOE for predicting oomycete effectors. POOE could achieve a highly accurate performance with an area under the precision-recall curve of 0.804 (area under the receiver operating characteristic curve = 0.893, accuracy = 0.874, precision = 0.777, recall = 0.684, and specificity = 0.936) in the fivefold cross-validation, considerably outperforming various combinations of popular machine learning algorithms and other commonly used sequence encoding schemes. A similar prediction performance was also observed in the independent test. Compared with the existing oomycete effector prediction methods, POOE provided very competitive and promising performance, suggesting that ProtTrans effectively captures rich protein semantic information and dramatically improves the prediction task. We anticipate that POOE can accelerate the identification of oomycete effectors and provide new hints to systematically understand the functional roles of effectors in plant–pathogen interactions. The web server of POOE is freely accessible at http://zzdlab.com/pooe/index.php. The corresponding source codes and data sets are also available at https://github.com/zzdlabzm/POOE.

**IMPORTANCE:**

In this work, we use the sequence representations from a pre-trained large protein language model (ProtTrans) as input and develop a Support Vector Machine-based method called POOE for predicting oomycete effectors. POOE could achieve a highly accurate performance in the independent test set, considerably outperforming existing oomycete effector prediction methods. We expect that this new bioinformatics tool will accelerate the identification of oomycete effectors and further guide the experimental efforts to interrogate the functional roles of effectors in plant-pathogen interaction.

## INTRODUCTION

Oomycetes are filamentous eukaryotic pathogens, including a large number of well-known plant pathogens such as *Phytophthora infestans*, *Hyaloperonospora arabidopsidis*, *Phytophthora ramorum*, and *Phytophthora sojae* (1). In general, oomycetes can be classified into four “crown” orders (i.e., Peronosporales, Pythiales, Albuginales, and Saprolegniales) based on a phylogenomic analysis of 65 oomycete species (2). One of the notorious plant diseases named late blight of potato, caused by the oomycete *Phytophthora infestans*, resulted in the Great Famine in Ireland (3). Another destructive species was *Phytophthora sojae*, which caused the annual soybean crop loss to billions of dollars (4). Even today, oomycete-related plant diseases can still lead to heavy economic losses worldwide (5, 6).

During the infection of oomycetes, a series of proteins called effectors are secreted into plants. Effectors perform their function by disturbing the host innate immunity to achieve their best benefit. Plants defend against oomycete infection by strengthening physical barriers, producing antimicrobial molecules, and initiating programmed cell death (7). Studying the effectors is instrumental in understanding the mechanisms of effector-triggered immunity (8) and developing effective disease control (7). According to the location of their targets, oomycete effectors can be categorized into at least two classes: apoplastic effectors and cytoplasmic effectors (9). Apoplastic effectors are secreted into the outside space of host cell membranes, while cytoplasmic effectors are translocated into the host cell (10). The difference between apoplastic and cytoplasmic effectors is that cytoplasmic effectors have conserved motifs following the signal peptide (11). Cytoplasmic effectors are roughly divided into two major groups. One is RXLR proteins which contain the RXLR motif, and the other class is Crinkler (CRN) proteins with two conserved motifs (i.e., LXLFLAK and HVLVVVP) in the N-terminus (12, 13). The RXLR motif, which is located in the N-terminal sequence after the secretion-related signal peptide (14), was reported to mediate the translocation into the host cell (15).

Both experimental and computational methods have been established to screen oomycete effectors. Traditional experimental approaches, including biochemical purification and map-based cloning, are widely used to identify oomycete effectors (14, 16). Recently, high-throughput experiments have also been used to screen candidate effectors. Although the proportion of potential effectors in an oomycete proteome is generally low (17), proteome-wide experimental validations of potential effectors are still not feasible. In this context, bioinformatics methods can be effectively used as the first step to narrow down the number of candidate effectors. For example, Wang et al. revealed that 45 *Phytophthora sojae* effectors significantly suppress programmed cell death through the functional validation of 169 computationally inferred oomycete effectors (18). Based on SignalP v2.0 (19), PexFinder was used to predict putative signal proteins on expressed sequence tags and obtained 142 putative secreted proteins (20). Through recursive BLAST search (21) based on the *Avr1b* (AARO5402) sequence and hidden Markov Model (HMM) (22) search of the RXLR domain, more than 700 candidate effector genes were identified from *Phytophthora sojae* and *Phytophthora ramorum* (23). Similarly, Goritschinig et al. used an HMM generated from the N-terminal conserved domains of previously identified effectors to screen 149 potential effectors in *Hyaloperonospora arabidopsidis* (24). The aforementioned computational methods depend highly on sequence similarity and translocation motif information. However, the sequence space of oomycete effectors is diverse. Thus, these routine bioinformatics strategies can hardly predict novel effectors without sharing any sequence homology with known effectors. Therefore, developing new bioinformatics methods independent of sequence homology and motif scanning is highly desirable.

Machine learning (ML) approaches may offer a promising alternative solution for oomycete effector prediction. With the rapid technical advance, ML-based methods have been prevalent in predicting tasks related to host–pathogen interaction (25, 26), including the detection of effectors from different pathogen proteomes. In particular, a series of ML algorithms, such as random forest (RF) (27), support vector machine (SVM) (28, 29), light gradient boosting machine (30), and deep learning methods (31), have been applied to predict bacterial type III secreted effectors, which are virulence proteins injected into host cells by Gram-negative bacteria. Recently, ML approaches were also proposed for fungal and oomycete effector prediction (32–36). EffectorP 1.0 and EffectorP 2.0 employed ML algorithms to identify fungal effectors from protein sequences, achieving >70% accuracy (34, 35). Their successor EffectorP 3.0 can predict whether a secreted fungal/oomycete protein is an apoplastic effector, a cytoplasmic effector, or a non-effector (33). Deepredeff is a deep learning classifier based on convolutional neural networks (CNNs), which could predict effectors of bacteria, fungi, and oomycetes (32). Trained on the N-terminus of effector sequences, an RF-based method EffectorO-ML was able to predict the oomycete effectors (36). Although the above methods have been elegantly used to accelerate the identification of oomycete effector proteins, they may still contain two limitations. First, the data of known oomycete effectors are insufficient, precluding the robust and unbiased performance estimation of the established predictive models. For instance, only 49, 85, and 88 oomycete effectors were used in training EffectorP 3.0, deepredeff, and EffectorO-ML, respectively. Second, the encoding strategies used in the above predictors are generally routine, indicating sufficient room for feature engineering improvement. In this context, we attempt to improve the oomycete effector prediction by compiling a more extensive data set in model training as well as seeking an optimal combination of ML methods and novel encoding schemes.

Natural language processing (NLP) focuses on automated text and language analysis, which has been rapidly developed in recent years (37). As a typical technique in NLP, the word embedding algorithm converted a word in a sentence, a paragraph, or an article into a distributed representation (38). Word2Vec is a representative word embedding model which uses a shallow two-layer neural network to learn word vectors. As an extension of Word2Vec, the Doc2Vec algorithm learned representations from the surrounding context words and the whole document (39). Such word/document embedding models have been used to process protein sequences and are further applied to protein bioinformatics tasks (40–42). Very recently, protein language models with unsupervised training inspired by NLP were investigated to extract features from a large volume of protein sequences (43–46). Interestingly, such pre-trained large protein models yield protein features containing intrinsic structural and functional properties of proteins (46, 47). One of these state-of-the-art protein language models is ProtTrans (44), which used the UniRef and Big Fantastic Database (BFD) data set as the corpus and employed two auto-regressive models and four auto-encoder models to generate the protein representations. ProtTrans has revealed its excellent performance in training downstream models for predicting biophysical features, such as secondary structure and protein sub-cellular location. Another representative protein language model is ESM-1b, which was trained on 86 billion amino acids across 250 million protein sequences spanning evolutionary diversity by Transformers (46). Undoubtedly, the features generated by these pre-trained large protein language models can be used for diverse protein bioinformatics prediction tasks, and their new applications are being rapidly explored (47–50).

**Fig 1:**
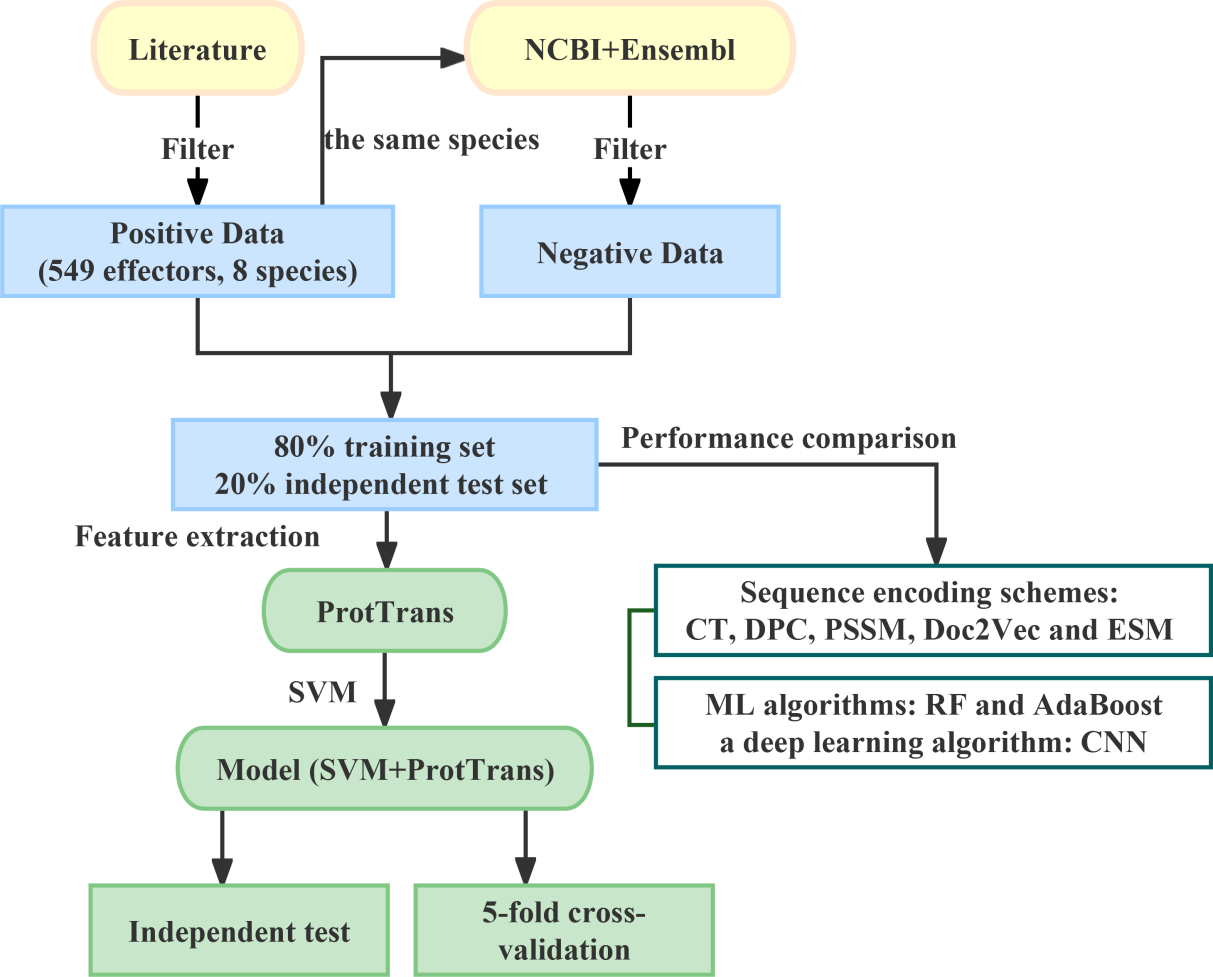
Workflow of our computational pipeline to predict oomycete effectors. First, we collected positive samples from the literature, downloaded proteomes for the eight species corresponding to positive samples from the NCBI and Ensembl databases, and conducted data filtration to obtain negative samples. The data set was further divided, in which 80% was taken as the training set for fivefold cross-validation and 20% as the independent test set. Second, we employed a pre-trained protein language model ProtTrans to extract sequence representations, allowing us to use SVM to predict effectors for the fivefold cross-validation and independent test. Finally, we compared our model with combinations of different encoding schemes and commonly used ML methods.

In this work, we developed a method called POOE based on the combination of ProtTrans and SVM to facilitate the screening of effector candidates (Fig. 1). POOE revealed high accuracy for classifying protein sequences as effectors or non-effectors through fivefold cross-validation and independent test. Moreover, we also benchmarked our prediction model against existing oomycete effector prediction methods. Finally, we made POOE accessible to the community through an online web server (http://zzdlab.com/pooe/index.php).

## MATERIALS AND METHODS

### Data collection and preprocessing

A total of 1,143 experimentally determined oomycete effectors were collected from the literature published before 1 January 2022 to prepare the positive samples. CD-HIT (51) with a 40% pairwise sequence identity cutoff was used to remove redundant sequences. Then, the species containing fewer than 10 effector sequences were deleted. As a result, 549 oomycete effectors from eight species were retained and regarded as positive samples (Table 1). To compile negative samples, we first collected all the eight proteome sequence data from the NCBI and Ensembl databases. Then, we removed protein sequences annotated as “effector” or containing RXLR, LXLFLAK, and HVLVVVP motifs. Moreover, we used CD-HIT to filter out redundant sequences at the 40% sequence identity cutoff, and sequences sharing 95% identity with positive samples were also discarded. In addition, proteins were selected with secretion signals predicted by SignalP 5.0 (52) and without transmembrane regions detected by TMHMM 2.0 (53). Finally, 3337 non-effector sequences were obtained. More details about the distributions of positive and negative samples in each species are available in Table 1.

**TABLE 1:** The positive and negative samples compiled in this study^*a*^

Species	Number of positive samples	Number of negative samples
*Phytophthora infestans*	180	373
*Phytophthora sojae*	129	569
*Phytophthora cactorum*	126	508
*Plasmopara viticola*	38	396
*Hyaloperonospora arabidopsidis*	33	304
*Bremia lactucae*	17	288
*Phytophthora nicotianae*	16	405
*Phytophthora capsici*	10	494
Total	549	3,337

^*a*^ The average sequence lengths in positive samples and negative samples are 194 ± 165 and 283 ± 288, respectively.

All these 549 oomycete effectors and putative 3,337 non-effectors were compiled as the original data set in this work. Considering that the number of effectors is much less than that of non-effectors within an oomycete proteome, a 1:3 ratio of positives to negatives was set to train and test the POOE predictor. Thus, we proportionally selected 1,670 negative samples from the species corresponding to the positive samples. Then, we further partitioned the original data set into two data sets: the training data (80%, 437 effectors and 1,338 non-effectors) and the independent data (20%, 112 effectors and 332 non-effectors). In order to fully demonstrate the performance of POOE, the data partitions based on the ratios of positives to negatives of 1:1 and 1:2 were also conducted to retrain and reassess the predictive model.

### Sequence-based encoding schemes

#### ProtTrans

ProtTrans is a series of pre-training protein language models, including two auto-regressive models and four auto-encoder models on 393 billion residues from UniRef and BFD. In our work, we downloaded an auto-encoder model called ProtT5 (ProtT5-XL-UniRef50) from https://github.com/agemagician/ProtTrans and installed it for local use. For each protein, the ProtT5 model, which was trained on the UniRef50 data set (54) by employing the T5 (Transfer Text-to-Text Transformer)-XL model, was used to generate a final representation of *L* × 1,024, where *L* is the length of the protein. We averaged over the length-dimension of the representations to derive fixed-size vectors (i.e., 1,024 dimensionality) for each protein.

#### ESM

Evolutionary Scale Modeling (ESM) is a repository provided by Meta which contains pre-trained protein language models to learn the relationship between sequence-structure-function. ESM-1b (46) is a large-scale Transformer model (650M parameters) trained on UniRef50 (54). We downloaded ESM from https://github.com/facebookresearch/esm and installed it for local use. By assigning the pre-trained model name to “ESM-1b” and the save method to “mean,” each protein was converted into a vector of 1,280 dimensionality by considering the embeddings averaged over the entire sequence.

#### Doc2Vec

As an unsupervised algorithm, Doc2Vec can obtain the vector representation of sentences, paragraphs, and documents. We chose non-redundant protein sequences with lengths between 30 and 5,000 amino acids from the SwissProt database (40, 55), using CD-HIT to remove redundant sequences (sequence identity ≤50%) (51). After these steps, 127,985 proteins were acquired as a corpus for the Doc2Vec model training. Referring to previous studies (40, 41, 56), we divided proteins into residue fragments (k-mers), using these fragments as words and complete sequences as sentences to train the Doc2Vec model. Here, we considered *k* = 3 and set the 32 dimensions for output vectors.

#### Position-specific scoring matrix (PSSM)

The PSSM profile was built through three-iteration PSI-BLAST searching with an E-value cut-off of 0.001 against the NCBI NR database (57). The scores of PSSM were normalized between 0 and 1, including the calculation of amino acid-specific composition (58). Let the length of one query sequence be *L*, and then the corresponding PSSM be an *L* × 20 scoring matrix (20 stands for the total number of residue types). In this work, we selected the first 200 N-terminal amino acids for calculation; thus, the final dimension of PSSM was 200 × 20.

#### Dipeptide composition (DPC)

DPC represents the compositions of two continuous amino acids (i.e., dipeptides) in the whole protein sequence, which was used to transform a protein into a 400-dimensional vector (59, 60).

#### Conjoint triad (CT)

To infer the CT encoding scheme, 20 amino acids were first divided into seven groups (AGV, C, DE, FILP, HNQW, KR, and MSTY) according to their side chains’ physicochemical properties. Then, the three consecutive amino acid group (i.e., CT) compositions within a protein sequence can be calculated (61). Consequently, a protein is represented by a 343-dimensional (7 × 7 × 7) vector.

### Parameter optimization for ML algorithms

This work compared the SVM-based POOE with two other traditional ML models [i.e., RF and adaptive boosting (AdaBoost)]. All the traditional ML models were implemented using the *scikit-learn* (https://scikit-learn.org) (62) package in Python. We used GridSearchCV for parameter optimization of different ML algorithms. In brief, GridSearchCV employs the Grid Search technique to find the optimal hyperparameters through fivefold cross-validation on the training data. In POOE, the kernel function was set to “rbf,” and the optimal C and gamma were 10 and 0.25, respectively. It is worth noting that the optimal ML parameters corresponding to different encoding schemes are usually different. Here, we took the parameter settings for ProtTrans in different ML algorithms as an example. In the RF model, the optimal n_estimators and max_depth were 420 and 8, respectively. Furthermore, we found that setting the parameters min_samples_split, min_samples_leaf, and max_features to 10, 5, and 32 yielded the best performance. In the AdaBoost model, the optimal number of trees and learning rate were 20 and 0.1, respectively. More details about the parameter search space and the optimal parameters of the three ML algorithms mentioned above are listed in Table S1.

We also compared the performance of POOE with a basic CNN model implemented using the Keras framework (https://keras.io/) in Python. The parameters of CNN were manually tuned. For ProtTrans, the CNN model consisted of four 1D convolutional layers, and each convolutional layer contained 16 channels. The kernel size of both convolutional layers was set to 7 with a stride of 1. The “ReLU” activation function was used to achieve non-linearity in these convolutional layers. The first convolutional layer was followed by a max pooling layer with a size of 3, and the second convolutional layer was connected to a global max pooling layer. These layers were then connected to three fully connected layers, containing 64, 16, and 1 neurons, respectively. The “Sigmoid” activation function was used to convert the final output into a probability score. Additionally, the dropout rate following fully connected layers was set to 0.5 to avoid overfitting. Moreover, we set the optimizer to “Adam” with a learning rate 0.0001. More details about the parameter optimization of CNN are also available in Table S1.

### Performance evaluation

In the fivefold cross-validation and the independent test, accuracy, precision, recall, and specificity were used to evaluate the prediction performance. These parameters are defined as follows:


$$Accuracy=\frac{TP+TN}{TP+TN+FP+FN}$$

$$Precision=\frac{TP}{TP+FP}$$

$$Recall=Sensitivity=\frac{TP}{TP+FN}$$

$$Specificity=\frac{TN}{TN+FP}$$

where TP, TN, FP, and FN represent the numbers of true positives, true negatives, false positives, and false negatives, respectively. To provide more comprehensively model assessment, the receiver operating characteristic (ROC) curve and precision-recall (PR) curve were plotted, and the corresponding areas under the ROC/PR curves (i.e., AUROC/AUPRC) were also employed to quantify the performance further. Generally, the closer the AUROC/AUPRC value is to 1, the better the performance of a prediction method is. Note that the PR curve and the corresponding AUPRC value seem more suitable for assessing models with imbalanced positive and negative samples.

## RESULTS AND DISCUSSION

### The performance of POOE

**Fig 2:**
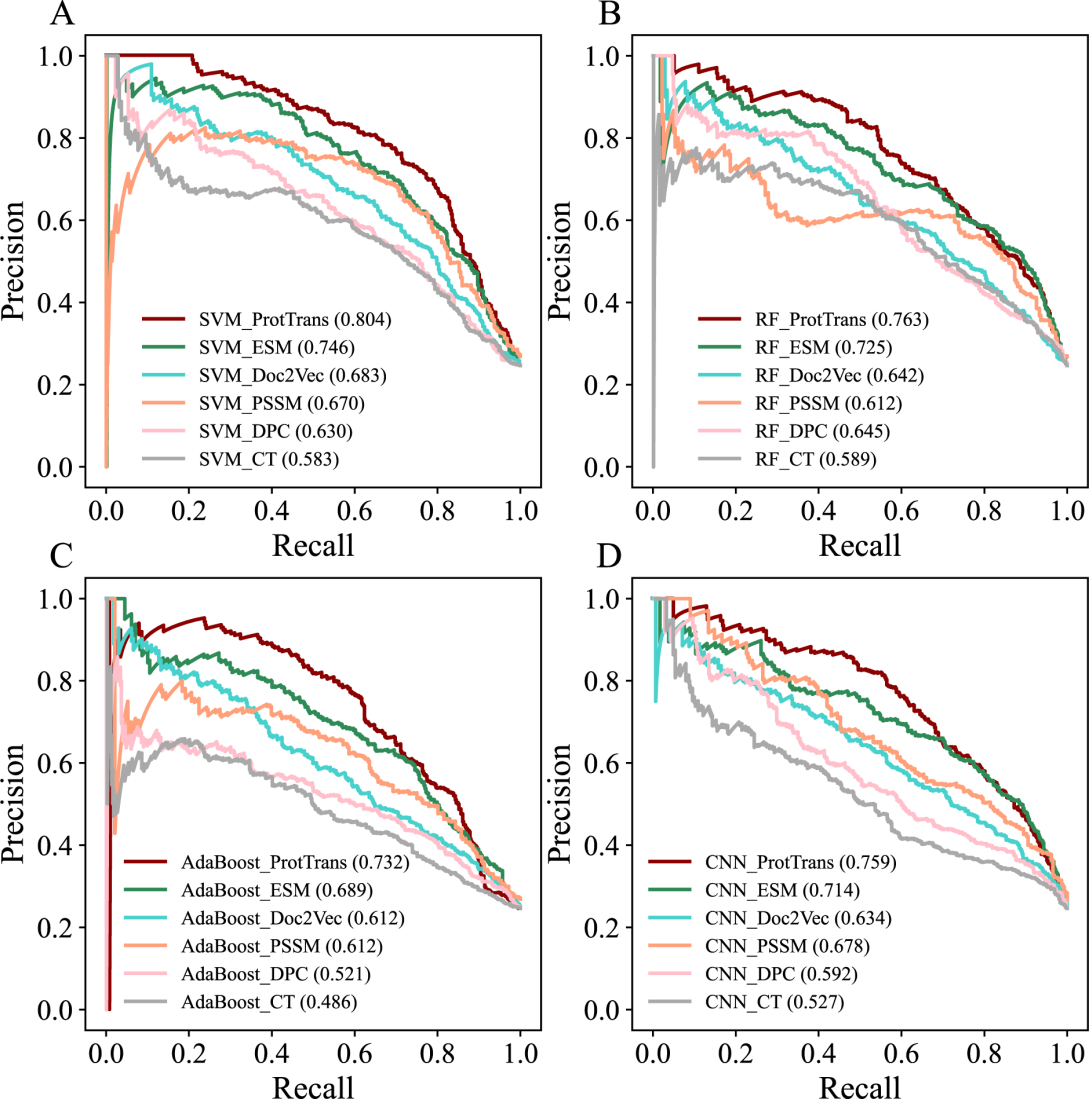
Performance of various classifiers on the fivefold cross-validation. We plotted precision-recall curves for the four machine learning models based on different sequence-based encoding schemes. Panels A, B, C, and D are the results of SVM, RF, AdaBoost, and CNN models. In each panel, the parameters in brackets denote the AUPRC values of the corresponding predictive models.

**Fig 3:**
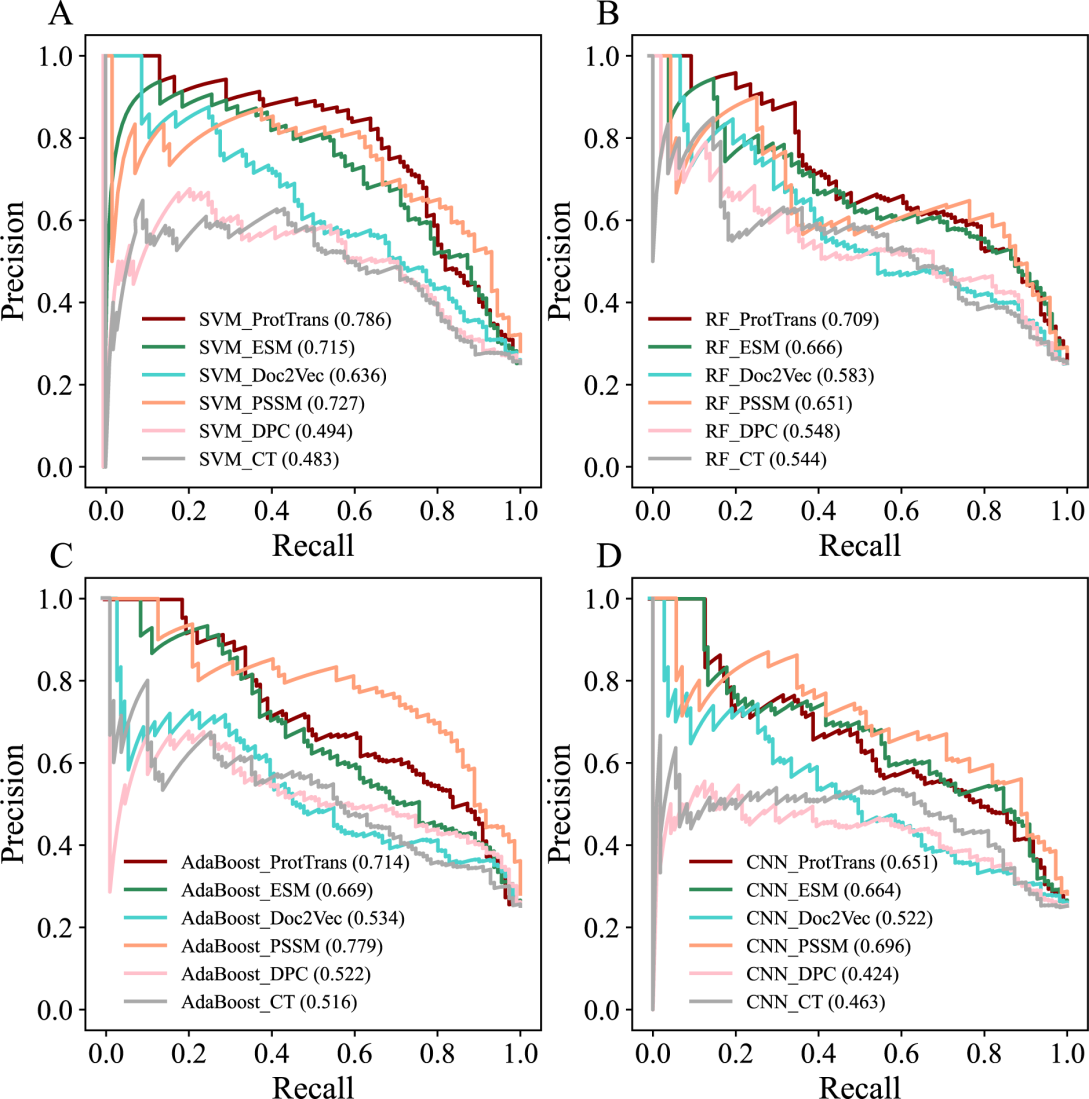
Performance of various classifiers on the independent test. We plotted precision-recall curves for the four machine learning models based on different sequence-based encoding schemes. Panels A, B, C, and D are the results of SVM, RF, AdaBoost, and CNN models. In each panel, the parameters in brackets denote the AUPRC values of the corresponding predictive models.

Here, we introduced a pre-trained language model called ProtTrans to convert protein sequences into feature vectors, allowing us to develop an SVM-based predictor called POOE to detect oomycete effectors. Through the fivefold cross-validation on the training data set with a ratio of 1:3 positives to negatives, POOE provided a highly accurate performance as the corresponding AUPRC value was 0.804 (AUROC = 0.893, accuracy = 0.874, precision = 0.777, recall = 0.684, specificity = 0.936) (Table 2; Fig. 2A). Likewise, POOE also performed excellently on the independent test with the corresponding AUPRC value was 0.786 (AUROC = 0.878, accuracy = 0.861, precision = 0.737, recall = 0.698, specificity = 0.916) (Table 3; Fig. 3A). The performance of POOE was also corroborated by the corresponding metrics when the model was trained and tested on the positives to negatives ratios of 1:1 and 1:2 (Fig. S1 and S2; Tables S2 to S5). To provide more rigorous performance comparisons, we also constructed two additional independent test sets named Additional test-38 and Additional test-29 to assess POOE. The positive samples of Additional test-38 consist of 38 effectors from previously left-out species in which the known effectors are <10, while the positive samples of Additional test-29 are 29 newly collected effectors from the literature published after 1 January 2022. The negative samples were constructed by following the same pipeline as we did in compiling training data, and the ratio of positives to negatives was set as 1:3. The value of AUPRC was 0.815 and 0. 920 for Additional test-38 and Additional test-29, respectively (Table S6), indicating that POOE can still obtain robust prediction performance on these two additional test sets (Table S6). Altogether, the success of the proposed POOE method suggests that the pre-trained ProtTrans model can capture rich protein semantic information, which can be effectively applied to distinguish effectors and non-effectors from the proteomes of oomycetes.

**TABLE 2:** Performance of various model combinations on the fivefold cross-validation

Method	AUPRC	AUROC	Accuracy	Precision	Recall	Specificity
SVM_CT	0.583	0.796	0.801	0.675	0.371	0.942
SVM_DPC	0.630	0.806	0.808	0.751	0.332	0.964
SVM_PSSM	0.670	0.847	0.820	0.790	0.451	0.956
SVM_Doc2Vec	0.683	0.833	0.830	0.746	0.471	0.948
SVM_ESM	0.746	0.875	0.852	0.726	0.643	0.921
SVM_ProtTrans	0.804	0.893	0.874	0.777	0.684	0.936
RF_CT	0.589	0.814	0.746	0.490	0.712	0.758
RF_DPC	0.645	0.816	0.810	0.623	0.581	0.885
RF_PSSM	0.612	0.825	0.776	0.608	0.469	0.889
RF_Doc2Vec	0.642	0.821	0.803	0.607	0.570	0.880
RF_ESM	0.725	0.882	0.837	0.720	0.554	0.930
RF_ProtTrans	0.763	0.885	0.852	0.754	0.590	0.937
AdaBoost_CT	0.486	0.738	0.777	0.623	0.238	0.953
AdaBoost_DPC	0.521	0.773	0.784	0.619	0.316	0.936
AdaBoost_PSSM	0.612	0.807	0.799	0.709	0.425	0.936
AdaBoost_Doc2Vec	0.612	0.801	0.805	0.681	0.391	0.940
AdaBoost_ESM	0.689	0.848	0.828	0.704	0.522	0.928
AdaBoost_ProtTrans	0.732	0.852	0.856	0.786	0.572	0.949
CNN_CT	0.527	0.751	0.785	0.632	0.302	0.942
CNN_DPC	0.592	0.791	0.794	0.640	0.371	0.932
CNN_PSSM	0.678	0.832	0.805	0.779	0.385	0.960
CNN_Doc2Vec	0.634	0.825	0.812	0.673	0.462	0.927
CNN_ESM	0.714	0.877	0.835	0.722	0.540	0.932
CNN_ProtTrans	0.759	0.884	0.856	0.786	0.572	0.949

**TABLE 3:** Performance of various model combinations on the independent test

Method	AUPRC	AUROC	Accuracy	Precision	Recall	Specificity
SVM_CT	0.483	0.751	0.775	0.594	0.343	0.920
SVM_DPC	0.494	0.761	0.774	0.628	0.254	0.949
SVM_PSSM	0.727	0.880	0.826	0.813	0.494	0.955
SVM_Doc2Vec	0.636	0.807	0.801	0.692	0.384	0.942
SVM_ESM	0.715	0.864	0.832	0.682	0.630	0.901
SVM_ProtTrans	0.786	0.878	0.861	0.737	0.698	0.916
RF_CT	0.544	0.775	0.733	0.480	0.688	0.749
RF_DPC	0.548	0.786	0.755	0.514	0.518	0.835
RF_PSSM	0.651	0.835	0.757	0.579	0.492	0.861
RF_Doc2Vec	0.583	0.784	0.755	0.514	0.496	0.842
RF_ESM	0.666	0.855	0.797	0.625	0.493	0.900
RF_ProtTrans	0.709	0.864	0.809	0.649	0.530	0.903
AdaBoost_CT	0.516	0.758	0.759	0.567	0.225	0.940
AdaBoost_DPC	0.522	0.797	0.758	0.537	0.286	0.917
AdaBoost_PSSM	0.779	0.897	0.812	0.775	0.467	0.948
AdaBoost_Doc2Vec	0.534	0.767	0.760	0.540	0.325	0.907
AdaBoost_ESM	0.669	0.829	0.797	0.628	0.484	0.903
AdaBoost_ProtTrans	0.714	0.854	0.812	0.664	0.520	0.911
CNN_CT	0.463	0.748	0.752	0.417	0.263	0.917
CNN_DPC	0.424	0.719	0.739	0.380	0.270	0.898
CNN_PSSM	0.696	0.854	0.772	0.611	0.336	0.942
CNN_Doc2Vec	0.522	0.735	0.758	0.552	0.361	0.892
CNN_ESM	0.664	0.840	0.805	0.656	0.491	0.911
CNN_ProtTrans	0.651	0.830	0.795	0.625	0.461	0.907

We further investigated the characteristics of misclassified sequences (i.e., false positives and false negatives) on the independent test set with the ratio of 1:3 positives to negatives. In the independent test set, 75 out of the 112 effectors were correctly predicted (i.e., true positives), whereas 37 were misclassified (i.e., false negatives). We examined the known sequence motifs (i.e., RXLR, LXLFLAK, and HVLVVVP) in the true positives and false negatives. The results showed that the proportion of true positives containing one of the known motifs is 57.3%. Comparatively, the corresponding proportion of false negatives is 35.1%. Therefore, our model tends to recognize sequences containing known sequence motifs as effectors. Regarding the predictive results of the 332 non-effectors, 21 sequences were misclassified as effectors (i.e., false positives). Interestingly, 16 out of the 21 false positives were found to have sequence similarity (BLAST E-value <1.0 × 10^−3^) with the 437 effectors. It should be emphasized that the characteristics of misclassified sequences are heavily relevant to the proposed encoding scheme and ML algorithm. On the other hand, the characteristics of misclassified sequences are also partly determined by the choice criteria of negative samples, since there is no existing golden standard for negative samples. For instance, sequences containing known motifs were filtered out in compiling negative samples, which may decrease the difficulty of our prediction task. We retrained POOE using negative samples without motif filtration, and the new model’s performance did decrease in both the fivefold cross-validation and independent test (Table S7). More interestingly, the observation that our model tends to recognize sequences containing known sequence motifs as effectors is less obvious.

It has been established that the secretion and translocation signals of oomycete effector proteins are located in the N-terminus. Thus, only N-terminal sequences, rather than full-length sequences, are often used for ML-based effector prediction. To investigate the effect of N-terminal sequence features on prediction performance, we retrained the SVM models based on ProtTrans using different lengths of N-terminal residues, including the sequence lengths of 30, 50, 70, 90, 110, 130, 150, and full length. As shown in Fig. S3, the AUPRC from the lengths of 50 to 70 increased rapidly and performed steadily when more N-terminal residues were taken into account, strongly confirming the secretion and translocation signals are located in the N-terminus. In our work, we decided to use full-length sequences for model training and real applications, since the robust performance can still be maintained.

### Comparison with different computational framework combinations

To benchmark the performance of POOE (i.e., SVM_ProtTrans), we compared SVM with the other two widely used ML algorithms (RF and AdaBoost) and one deep learning method (CNN) and ProtTrans against five commonly used encoding schemes (CT, DPC, PSSM, Doc2Vec, and ESM). The results showed that the combination of SVM and ProtTrans achieved the best performance (Fig. 2; Fig. S1; Table 2). Regarding different prediction algorithms, SVM is the most suitable for predicting oomycete effectors when using different encoding schemes as input, followed by RF, CNN, and AdaBoost. For instance, using ProtTrans as input, SVM (AUPRC = 0.804) outperformed RF (AUPRC = 0.763), CNN (AUPRC = 0.759), and AdaBoost (AUPRC = 0.732) in the fivefold cross-validation (Fig. 2; Fig. S1; Table 2). Likewise, we observed similar performance ranks in the independent test (Fig. 3; Fig. S2; Table 3). In addition, we obtained a similar trend for different ML algorithms when the positives to negatives ratio was set to 1:1 and 1:2 (Tables S2 to S5).

Regarding the encoding schemes under investigation, ProtTrans was the most informative in combining with different ML algorithms. In the context of SVM-based models, ProtTrans achieved the best performance, followed by the other two NLP-based embeddings (ESM and Doc2Vec) and three conventional encoding schemes (PSSM, DPC, and CT) (Table 2). Comparatively, PSSM was better than DPC or CT, since it could capture evolutionary information within protein sequences, which is in line with its performance in other protein classification tasks. Regarding the performance of three NLP-based embeddings, it is also interesting to emphasize that ProtTrans reveals slightly better performance than ESM, but both outperformed Doc2Vec considerably. The inferior performance of Doc2Vec could be ascribed to the limited size of the corpus and the less informative model caused by shallow learning to infer the protein embeddings. Considering that pre-trained protein language models are increasingly available now, we further benchmarked ProtTrans against two popular models named TAPE and SeqVec. The performance of fivefold cross-validation and independent tests consistently showed that ProtTrans outperformed TAPE and SeqVec under different ML algorithms, further demonstrating the superiority of ProtTrans (Table S8). Taken together, the benchmark experiments mentioned above confirmed that the pre-trained large protein language model ProtTrans could convert protein sequences into feature vectors with rich semantic information, which was very suitable for distinguishing effectors and non-effectors in oomycete proteomes. Moreover, the conventional ML algorithm SVM was suitable for dealing with the feature vectors extracted from ProtTrans. The results of CNN were inferior to those of RF and SVM, which indicated that deep learning might be less potent in dealing with data sets with limited size.

### Comparison with existing oomycete effector prediction methods

To benchmark our method, we first compared POOE with three existing oomycete effector predictors [i.e., EffectorO (36), EffectorP3.0 (33), and deepredeff (32)] using the independent test data set. As the key component of EffectorO, EffectorO-ML is an RF classifier using the N-terminal residue properties as input. We uploaded the independent test data set to EffectorO-ML (https://effectoro.onrender.com/) to obtain the prediction results. EffectorP3.0 was developed for predicting fungal and oomycete effectors simultaneously. Similarly, the independent test data set was uploaded to the web server of EffectorP3.0 (https://effectorp.csiro.au/) to predict oomycete effectors. Deepredeff contains four models, the CNN-LSTM model, CNN-GRU model, LSTM-Embedding model, and GRU-Embedding model, to predict effectors of bacteria, fungi, and oomycetes. We chose the CNN-LSTM model, which was reported to perform relatively well in predicting oomycete effectors. The corresponding model was downloaded to predict the independent test data set in our local machine. Table 4 indicated that our POOE model could dramatically outperform EffectorO, EffectorP3.0, and deepredeff in most performance metrics. For instance, POOE revealed an approximately 0.4 increase in AUPRC compared to the three existing predictors (Table 4).

**TABLE 4:** Performance comparison of our POOE model with existing prediction methods

Method	AUPRC	AUROC	Accuracy	Precision	Recall	Specificity
POOE (SVM_ProtTrans)	0.786	0.878	0.861	0.737	0.698	0.916
EffectorO	0.372	0.654	0.495	0.310	0.812	0.389
EffectorP 3.0	0.402	0.668	0.559	0.332	0.741	0.497
Deepredeff	0.383	0.587	0.703	0.384	0.295	0.840
BLAST	0.398	0.726	0.727	0.473	0.714	0.732
Motif scanning	0.565	0.745	0.529	0.326	0.812	0.434
Motif scanning (without motif filtration^*a*^)	0.451	0.674	0.471	0.298	0.812	0.355

^*a*^ We reconstructed negative samples without implementing the motif filtration. Based on the newly compiled negative samples, we reassessed the performance of the motif scanning method.

We further benchmarked POOE against EffectorO, EffectorP3.0, and deepredeff on Additional test-38 and Additional test-29. As shown in Table S6, POOE performed better than the three existing methods. We also used the EffectorO method’s data to retrain the POOE model (i.e., SVM + ProtTrans), which allowed a more comprehensive comparison between these two methods. The results confirmed that POOE is superior to EffectorO (Table S6). Subsequently, we used POOE and EffectorO to conduct proteome-wide identification of effectors on *Phytophthora parasitica*. After the initial filtering, 1,515 out of 22,979 proteins in *Phytophthora parasitica* were predicted as secreted proteins without transmembrane regions and were further submitted to POOE and EffectorO. The predictive threshold at a specificity control at 89.8% [i.e., false positive rate (FPR) control at 10.2%] was provided by EffectorO. To ensure a fair comparison between POOE and EffectorO, we also reported the POOE results at the FPR control of 10.2%, and the results showed that 324 proteins in *Phytophthora parasitica* were predicted as effectors by POOE. Interestingly, one known *Phytophthora parasitica* effector in the 1,515 secreted proteins was successfully predicted. Based on the same FPR control, 239 of the 406 effectors predicted by EffectorO overlapped with the POOE’s results (Fig. S4). The consistent predictions of POOE and EffectorO indicate a general reliability of POOE indirectly. Moreover, these two predictors also identified unique effector candidates respectively, suggesting that they are complementary to some extent. Therefore, different predictors should be jointly used to maximize the prediction performance in real applications.

### Comparison with sequence/motif searching-based effector identification strategy

Considering oomycete effectors may share sequence similarity or contain some specific sequence motifs, classical sequence and motif searching have been widely used to recognize potential new oomycete effectors in real applications. Thus, it is very natural to compare POOE further with these two routine effector detection strategies. We compared POOE with BLAST (57), the most frequently used sequence similarity searching tool. To this end, the whole effector sequences from the training data were used to build the standalone BLAST database, while the sequences from the independent test data set were used as query proteins. For each query, the top hit was kept, and the corresponding E-value was defined as the BLAST prediction score. As a default setting, the query was predicted as an effector if the E-value was less than 1.0 × 10^−3^; otherwise, it was predicted as a non-effector. The results showed that BLAST can achieve reasonable performance comparable to the three existing oomycete effector predictors, but it is still inferior to POOE considerably (Table 4). To implement the motif searching-based effector identification, the FIMO program (63) was used to scan the sequences in the independent test data set. As a component of the MEME package, FIMO scans DNA or protein sequences for provided motifs and computes the *P*-value of each hit. If the *P*-value was less than 1.0 × 10^−3^, the corresponding hit would be considered as a true motif. In case multiple hits were identified, only the most significant one was kept, and the corresponding *P*-value was regarded as the output score. We employed FIMO to search the three most common motifs: RXLR, LXLFLAK, and HVLVVVP. We observed that the motif scanning strategy performed reasonably well, although it was worse than POOE (Table 4), indicating that the motif information alone was informative for detecting effectors. However, we noticed that the good performance of motif searching could be a biased result caused by our negative sampling strategy. When the motif filtration was not used in compiling negative samples, we revisited the motif searching method, and the corresponding performance decreased (Table 4).

### Implementation of POOE

To facilitate the broader research community, we developed an online web server as the implementation of POOE, which can be freely accessible at http://zzdlab.com/pooe/index.php. Note that POOE was optimized for processing proteins with sequence lengths of more than 20 amino acids. The maximal number of sequences per job is 200. Five predictive models generated in the fivefold cross-validation with the positives to negatives ratio of 1:3 are jointly used to conduct prediction in the web server, and the final prediction score of a query protein is the average score generated from the five different models. Two thresholds (i.e., specificity controls at 97% and 93%, corresponding to predictive scores of 0.70 and 0.50, respectively) are provided to determine whether a protein is an oomycete effector. POOE offers three ways, namely, by job name, job ID, and submission date, for users to query their submitted jobs. As such, the job name should be provided by users when submitting their sequences. After the submission task is completed, the raw prediction scores and whether they are predicted as oomycete effectors will be returned to the result page. We have also made the source codes and the data sets used in this work freely downloadable on our server and GitHub (https://github.com/zzdlabzm/POOE).

### Conclusions

In this work, we developed a novel ProtTrans embedding-based SVM classifier termed POOE for predicting oomycete effectors. Benchmarking experiments indicated that our POOE outperformed computational framework combinations of the other five sequence encodings and three widely used ML algorithms. Moreover, the proposed method surpassed several existing oomycete effector predictors. We observed that the embedding generated by the ProtTrans language model could capture rich semantic information regarding protein sequence-structure-function relationships and improve downstream prediction tasks. To sum up, we expect that this new bioinformatics tool will accelerate the identification of oomycete effectors and further guide the experimental efforts to interrogate the functional roles of effectors in plant–pathogen interaction.

## SUPPLEMENTAL MATERIAL

Supplemental material msystems.01004-23-s0001.docx Supplemental figures and tables.

ASM does not own the copyrights to Supplemental Material that may be linked to, or accessed through, an article. The authors have granted ASM a non-exclusive, world-wide license to publish the Supplemental Material files. Please contact the corresponding author directly for reuse.
