# Supplementary material for: POOE: predicting oomycete effectors based on a pre-trained large protein language model
Source: mSystems. 2023 Dec 11;9(1):e01004-23. doi: 10.1128/msystems.01004-23 (PMC10804963; doi:10.1128/msystems.01004-23)
Supplement: Supplemental material — Supplemental figures and tables. [file msystems.01004-23-s0001.docx]

**Supplementary information**

**POOE: Predicting Oomycete Offectors Based on a Pre-trained Large Protein Language Model**

*Miao Zhao, Chenping Lei, Kewei Zhou, Yan Huang,* *Chen Fu, Shiping Yang** *and Ziding Zhang**

*Corresponding authors: Dr. Shiping Yang (E-mail: [shipingyang@cau.edu.cn](mailto:shipingyang@cau.edu.cn)) and Dr. Ziding Zhang (E-mail: zidingzhang@cau.edu.cn)

The supplementary material contains:

1. Supplementary Tables S1-8
2. Supplementary Figures S1-4

Table S1. Optimization and setting of model parameters in different ML models.

| Model^a,b^ | Parameter | Parameter search space^c^ | CT | DPC | PSSM | Doc2Vec | ESM | ProtTrans | TAPE | SeqVec |
| --- | --- | --- | --- | --- | --- | --- | --- | --- | --- | --- |
| AdaBoost | n_estimators | R: 10-200 I: 10 | 140 | 180 | 130 | 70 | 160 | 20 | 50 | 110 |
|  | learning_rate | [0.01, 0.05, 0.1, 0.2, 0.3, 0.4] | 0.1 | 0.2 | 0.3 | 0.4 | 0.1 | 0.1 | 0.3 | 0.3 |
|  | max_depth | [3, 4, 5, 6, 7, 8, 9] | 7 | 7 | 6 | 8 | 7 | 3 | 9 | 9 |
|  | min_samples_split | [10, 20, 30, 40, 50, 60, 70] | 50 | 50 | 70 | 40 | 60 | 10 | 30 | 70 |
| RF | n_estimators | R: 100-500 I: 10 | 390 | 180 | 480 | 410 | 480 | 420 | 270 | 200 |
|  | max_depth | [3, 4, 5, 6, 7, 8, 9] | 9 | 9 | 8 | 9 | 8 | 8 | 9 | 9 |
|  | min_samples_split | [10, 20, 30, 40, 50, 60, 70, 80, 90, 100] | 10 | 10 | 10 | 10 | 10 | 10 | 10 | 10 |
|  | min_samples_leaf | [5, 10, 15, 20, 25, 30, 35, 40, 45, 50] | 45 | 10 | 20 | 10 | 20 | 5 | 5 | 5 |
|  | max_features | R: 3-40 I: 1 | 10 | 20 | 38 | 3 | 38 | 32 | 28 | 26 |
|  | random_state | [10] | 10 | 10 | 10 | 10 | 10 | 10 | 10 | 10 |
|  | class_weight | [‘balanced’] | balanced | balanced | balanced | balanced | balanced | balanced | balanced | balanced |
| SVM | C | [0.01, 0.1, 1, 10, 100] | 100 | 100 | 1 | 1 | 100 | 10 | 10 | 10 |
|  | gamma | [2^-10^, 2^-9^, 2^-8^, 2^-7^, 2^-6^, 2^-5^, 2^-4^, 2^-3^, 2^-2^, 2^-1^, 2^0^, 2^1^, 2^2^, 2^3^, 2^4^, 2^5^] | 2^-1^ | 2^-2^ | -- | 2^-5^ | -- | 2^-2^ | -- | 2^-5^ |
|  | degree | [2, 3, 4, 5] | -- | -- | 2 | -- | 4 | -- | 3 | -- |
|  | kernel | [‘rbf’, ‘poly’] | rbf | rbf | poly | rbf | poly | rbf | poly | rbf |
| CNN | learning_rate | [0.0001, 0.001, 0.01, 0.1] | 0.001 | 0.001 | 0.0001 | 0.0001 | 0.0001 | 0.0001 | 0.0001 | 0.0001 |
|  | epochs | R:1-100 I:1 | 14 | 30 | 20 | 60 | 30 | 40 | 20 | 20 |
|  | batch_size | [8, 16, 32, 64] | 8 | 32 | 16 | 16 | 16 | 16 | 16 | 16 |
|  | 1D convolutional layers | [2,3,4] | 3 | 3 | 3 | 2 | 4 | 3 | 4 | 4 |
|  | kernel size | [3,5,7] | 7 | 7 | 5 | 5 | 5 | 7 | 7 | 7 |
|  | optimizers | [Adam] | Adam | Adam | Adam | Adam | Adam | Adam | Adam | Adam |
|  | dropout_rate | [0.5] | 0.5 | 0.5 | 0.5 | 0.5 | 0.5 | 0.5 | 0.5 | 0.5 |
|  | tf.random.set_seed | [10] | 10 | 10 | 10 | 10 | 10 | 10 | 10 | 10 |

^a^ We used GridSearchCV implemented in scikit-learn for the parameter optimization of different machine learning algorithms. For AdaBoost, we selected DecisionTreeClassifier as the base_estimator and parameterized the n_estimators and learning_rate first. Then, we adjusted the max_depth and min_samples_split in the decision tree with the fixed values of n_estimators and learning_rate. For RF, the values of parameters were determined in order of n_estimators, max_depth, min_samples_split, min_samples_leaf, and max_features, while the random_state was set as 10 to ensure consistent results from each run. For SVM, the values of C, gamma (kernel = ‘rbf’) and degree (kernel = ‘poly’) were set and the optimal parameters of the model were selected. ‘--’ means this parameter is not avaiable for current parameter combinations.

^b^The parameters of CNN were manually tuned. First, we determined the learning rate by monitoring the loss curve. Second, the value of batch size depended on AUPRC and AUROC. Third, set epochs equal to 100, and select the epoch value according to the overall situation of the model loss curve and early stopping. Kernel size and convolutional layers were determined according to AUPRC and AUROC.

^c^ R represents the range and I represents the interval.

Table S2. Performance of various computational framework combinations on the 5-fold cross-validation using the 1:1 ratio of positive to negative samples.

| Method | AUPRC | AUROC | Accuracy | Precision | Recall | Specificity |
| --- | --- | --- | --- | --- | --- | --- |
| SVM_CT | 0.817 | 0.814 | 0.776 | 0.785 | 0.769 | 0.782 |
| SVM_DPC | 0.811 | 0.811 | 0.759 | 0.761 | 0.765 | 0.752 |
| SVM_PSSM | 0.858 | 0.863 | 0.792 | 0.780 | 0.842 | 0.736 |
| SVM_Doc2Vec | 0.820 | 0.826 | 0.757 | 0.782 | 0.724 | 0.792 |
| SVM_ESM | 0.847 | 0.872 | 0.811 | 0.802 | 0.834 | 0.787 |
| SVM_ProtTrans | 0.878 | 0.885 | 0.828 | 0.842 | 0.814 | 0.843 |
| RF_CT | 0.819 | 0.818 | 0.755 | 0.745 | 0.787 | 0.722 |
| RF_DPC | 0.814 | 0.817 | 0.748 | 0.747 | 0.762 | 0.734 |
| RF_PSSM | 0.815 | 0.829 | 0.740 | 0.838 | 0.626 | 0.866 |
| RF_Doc2Vec | 0.829 | 0.816 | 0.751 | 0.771 | 0.724 | 0.778 |
| RF_ESM | 0.849 | 0.865 | 0.805 | 0.836 | 0.767 | 0.845 |
| RF_ProtTrans | 0.860 | 0.871 | 0.808 | 0.856 | 0.747 | 0.870 |
| AdaBoost_CT | 0.778 | 0.777 | 0.716 | 0.723 | 0.715 | 0.718 |
| AdaBoost_DPC | 0.766 | 0.781 | 0.712 | 0.714 | 0.722 | 0.701 |
| AdaBoost_PSSM | 0.808 | 0.802 | 0.742 | 0.742 | 0.780 | 0.699 |
| AdaBoost_Doc2Vec | 0.769 | 0.772 | 0.705 | 0.702 | 0.729 | 0.681 |
| AdaBoost_ESM | 0.823 | 0.828 | 0.768 | 0.780 | 0.756 | 0.780 |
| AdaBoost_ProtTrans | 0.822 | 0.847 | 0.788 | 0.808 | 0.765 | 0.813 |
| CNN_CT | 0.577 | 0.553 | 0.518 | 0.517 | 0.780 | 0.248 |
| CNN_DPC | 0.559 | 0.501 | 0.502 | 0.509 | 0.601 | 0.400 |
| CNN_PSSM | 0.896 | 0.881 | 0.775 | 0.771 | 0.813 | 0.732 |
| CNN_Doc2Vec | 0.792 | 0.778 | 0.703 | 0.716 | 0.688 | 0.718 |
| CNN_ESM | 0.837 | 0.858 | 0.806 | 0.824 | 0.787 | 0.826 |
| CNN_ProtTrans | 0.871 | 0.879 | 0.789 | 0.861 | 0.697 | 0.884 |

Table S3. Performance of various computational framework combinations on the independent test using the 1:1 ratio of positive to negative samples.

| Method | AUPRC | AUROC | Accuracy | Precision | Recall | Specificity |
| --- | --- | --- | --- | --- | --- | --- |
| SVM_CT | 0.818 | 0.812 | 0.772 | 0.764 | 0.744 | 0.797 |
| SVM_DPC | 0.809 | 0.802 | 0.735 | 0.706 | 0.748 | 0.725 |
| SVM_PSSM ^a^ | 0.895 | 0.871 | 0.817 | 0.846 | 0.830 | 0.800 |
| SVM_Doc2Vec | 0.825 | 0.825 | 0.740 | 0.726 | 0.713 | 0.764 |
| SVM_ESM | 0.882 | 0.897 | 0.814 | 0.779 | 0.841 | 0.790 |
| SVM_ProtTrans | 0.885 | 0.883 | 0.802 | 0.776 | 0.812 | 0.793 |
| RF_CT | 0.826 | 0.837 | 0.775 | 0.737 | 0.810 | 0.745 |
| RF_DPC | 0.819 | 0.830 | 0.735 | 0.695 | 0.775 | 0.701 |
| RF_PSSM ^a^ | 0.854 | 0.831 | 0.675 | 0.819 | 0.551 | 0.839 |
| RF_Doc2Vec | 0.801 | 0.801 | 0.706 | 0.690 | 0.678 | 0.732 |
| RF_ESM | 0.865 | 0.882 | 0.797 | 0.792 | 0.769 | 0.822 |
| RF_ProtTrans | 0.864 | 0.878 | 0.785 | 0.797 | 0.724 | 0.838 |
| AdaBoost_CT | 0.813 | 0.825 | 0.741 | 0.720 | 0.732 | 0.749 |
| AdaBoost_DPC | 0.802 | 0.821 | 0.728 | 0.707 | 0.718 | 0.737 |
| AdaBoost_PSSM ^a^ | 0.910 | 0.874 | 0.752 | 0.790 | 0.770 | 0.729 |
| AdaBoost_Doc2Vec | 0.812 | 0.802 | 0.692 | 0.665 | 0.691 | 0.692 |
| AdaBoost_ESM | 0.864 | 0.889 | 0.789 | 0.779 | 0.767 | 0.809 |
| AdaBoost_ProtTrans | 0.851 | 0.864 | 0.770 | 0.753 | 0.757 | 0.781 |
| CNN_CT | 0.592 | 0.613 | 0.494 | 0.489 | 0.781 | 0.241 |
| CNN_DPC | 0.734 | 0.500 | 0.494 | 0.281 | 0.600 | 0.400 |
| CNN_PSSM ^a^ | 0.887 | 0.841 | 0.726 | 0.767 | 0.751 | 0.693 |
| CNN_Doc2Vec | 0.699 | 0.726 | 0.632 | 0.599 | 0.652 | 0.614 |
| CNN_ESM | 0.858 | 0.870 | 0.779 | 0.766 | 0.767 | 0.790 |
| CNN_ProtTrans | 0.857 | 0.857 | 0.764 | 0.774 | 0.703 | 0.817 |

^a^ Considering that the PSSM profiles of some sequences are not available, only 74 positive samples and 56 negative samples in the independent test dataset were used for performance assessment. This may inevitably lead to the performance overestimation of the PSSM encoding scheme.

Table S4. Performance of various computational framework combinations on the 5-fold cross-validation using the 1:2 ratio of positive to negative samples.

| Method | AUPRC | AUROC | Accuracy | Precision | Recall | Specificity |
| --- | --- | --- | --- | --- | --- | --- |
| SVM_CT | 0.694 | 0.806 | 0.776 | 0.734 | 0.527 | 0.903 |
| SVM_DPC | 0.701 | 0.811 | 0.781 | 0.733 | 0.550 | 0.898 |
| SVM_PSSM | 0.739 | 0.845 | 0.804 | 0.750 | 0.679 | 0.873 |
| SVM_Doc2Vec | 0.741 | 0.840 | 0.788 | 0.757 | 0.547 | 0.911 |
| SVM_ESM | 0.782 | 0.881 | 0.816 | 0.732 | 0.718 | 0.866 |
| SVM_ProtTrans | 0.833 | 0.896 | 0.841 | 0.781 | 0.732 | 0.896 |
| RF_CT | 0.712 | 0.825 | 0.780 | 0.673 | 0.676 | 0.833 |
| RF_DPC | 0.723 | 0.824 | 0.786 | 0.688 | 0.667 | 0.847 |
| RF_PSSM | 0.725 | 0.840 | 0.771 | 0.726 | 0.580 | 0.878 |
| RF_Doc2Vec | 0.725 | 0.825 | 0.773 | 0.687 | 0.599 | 0.861 |
| RF_ESM | 0.778 | 0.883 | 0.816 | 0.754 | 0.676 | 0.888 |
| RF_ProtTrans | 0.808 | 0.891 | 0.831 | 0.791 | 0.680 | 0.908 |
| AdaBoost_CT | 0.616 | 0.746 | 0.743 | 0.678 | 0.450 | 0.891 |
| AdaBoost_DPC | 0.629 | 0.767 | 0.752 | 0.687 | 0.489 | 0.887 |
| AdaBoost_PSSM | 0.663 | 0.765 | 0.726 | 0.648 | 0.518 | 0.843 |
| AdaBoost_Doc2Vec | 0.697 | 0.809 | 0.770 | 0.707 | 0.543 | 0.885 |
| AdaBoost_ESM | 0.764 | 0.844 | 0.800 | 0.756 | 0.601 | 0.901 |
| AdaBoost_ProtTrans | 0.773 | 0.856 | 0.800 | 0.727 | 0.653 | 0.875 |
| CNN_CT | 0.589 | 0.734 | 0.708 | 0.688 | 0.248 | 0.943 |
| CNN_DPC | 0.627 | 0.776 | 0.734 | 0.659 | 0.439 | 0.884 |
| CNN_PSSM | 0.749 | 0.832 | 0.757 | 0.789 | 0.438 | 0.935 |
| CNN_Doc2Vec | 0.650 | 0.767 | 0.745 | 0.675 | 0.468 | 0.885 |
| CNN_ESM | 0.766 | 0.873 | 0.827 | 0.758 | 0.714 | 0.884 |
| CNN_ProtTrans | 0.774 | 0.857 | 0.812 | 0.807 | 0.583 | 0.929 |

Table S5. Performance of various computational framework combinations on the independent test using the 1:2 ratio of positive to negative samples.

| Method | AUPRC | AUROC | Accuracy | Precision | Recall | Specificity |
| --- | --- | --- | --- | --- | --- | --- |
| SVM_CT | 0.605 | 0.768 | 0.741 | 0.635 | 0.440 | 0.882 |
| SVM_DPC | 0.638 | 0.780 | 0.749 | 0.634 | 0.501 | 0.865 |
| SVM_PSSM ^a^ | 0.861 | 0.891 | 0.844 | 0.815 | 0.762 | 0.894 |
| SVM_Doc2Vec | 0.704 | 0.812 | 0.766 | 0.676 | 0.510 | 0.885 |
| SVM_ESM | 0.754 | 0.858 | 0.810 | 0.697 | 0.714 | 0.854 |
| SVM_ProtTrans | 0.815 | 0.882 | 0.822 | 0.727 | 0.709 | 0.876 |
| RF_CT | 0.626 | 0.794 | 0.752 | 0.608 | 0.619 | 0.813 |
| RF_DPC | 0.650 | 0.789 | 0.740 | 0.586 | 0.623 | 0.795 |
| RF_PSSM ^a^ | 0.814 | 0.876 | 0.774 | 0.746 | 0.614 | 0.872 |
| RF_Doc2Vec | 0.659 | 0.789 | 0.758 | 0.622 | 0.611 | 0.827 |
| RF_ESM | 0.750 | 0.850 | 0.802 | 0.717 | 0.623 | 0.885 |
| RF_ProtTrans | 0.765 | 0.854 | 0.795 | 0.712 | 0.598 | 0.887 |
| AdaBoost_CT | 0.572 | 0.772 | 0.733 | 0.616 | 0.436 | 0.872 |
| AdaBoost_DPC | 0.610 | 0.766 | 0.721 | 0.583 | 0.429 | 0.858 |
| AdaBoost_PSSM ^a^ | 0.798 | 0.843 | 0.729 | 0.691 | 0.526 | 0.854 |
| AdaBoost_Doc2Vec | 0.697 | 0.806 | 0.755 | 0.649 | 0.505 | 0.872 |
| AdaBoost_ESM | 0.721 | 0.831 | 0.771 | 0.684 | 0.516 | 0.890 |
| AdaBoost_ProtTrans | 0.784 | 0.846 | 0.797 | 0.707 | 0.619 | 0.880 |
| CNN_CT | 0.556 | 0.746 | 0.708 | 0.435 | 0.230 | 0.931 |
| CNN_DPC | 0.625 | 0.785 | 0.716 | 0.465 | 0.385 | 0.871 |
| CNN_PSSM ^a^ | 0.696 | 0.832 | 0.720 | 0.600 | 0.397 | 0.918 |
| CNN_Doc2Vec | 0.626 | 0.708 | 0.724 | 0.602 | 0.446 | 0.853 |
| CNN_ESM | 0.729 | 0.835 | 0.792 | 0.685 | 0.642 | 0.862 |
| CNN_ProtTrans | 0.671 | 0.802 | 0.760 | 0.681 | 0.467 | 0.897 |

^a^ Considering that the PSSM profiles of some sequences are not available, only 73 positive samples and 119 negative samples in the independent test dataset were used for performance assessment. This may inevitably lead to the performance overestimation of the PSSM encoding scheme.

Table S6. Performance of POOE and existing effector prediction methods on Additional test-38，Additional test-29 and EffectorO’s data.

|  | AUPRC | AUROC | Accuracy | Precision | Recall | Specificity |
| --- | --- | --- | --- | --- | --- | --- |
| *Additional test-38* |  |  |  |  |  |  |
| POOE | 0.815 | 0.909 | 0.882 | 0.810 | 0.689 | 0.946 |
| EffectorO | 0.748 | 0.818 | 0.578 | 0.357 | 0.862 | 0.483 |
| EffectorP 3.0 | 0.682 | 0.837 | 0.698 | 0.448 | 0.897 | 0.632 |
| deepredreff | 0.627 | 0.750 | 0.819 | 0.633 | 0.655 | 0.874 |
| *Additional test-29* |  |  |  |  |  |  |
| POOE | 0.920 | 0.934 | 0.948 | 0.953 | 0.834 | 0.986 |
| EffectorO | 0.532 | 0.723 | 0.566 | 0.337 | 0.763 | 0.500 |
| EffectorP 3.0 | 0.554 | 0.783 | 0.671 | 0.419 | 0.816 | 0.623 |
| deepredreff | 0.391 | 0.622 | 0.691 | 0.364 | 0.316 | 0.816 |
| *EffectorO’s data* |  |  |  |  |  |  |
| POOE | 0.997 | 0.997 | 0.955 | 0.976 | 0.932 | 0.977 |
| EffectorO^a^ | - | 0.890 | 0.843 | - | 0.870 | 0.830 |

^a^ EffectorO’s results were directly retrieved from the original literature of EffectorO. “-” stands for the corresponding result in the original literature is not available.

Table S7. Model performance based on different negative sample constructions using the 1:3 ratio of positive to negative samples.

|  | AUPRC | AUROC | Accuracy | Precision | Recall | Specificity |
| --- | --- | --- | --- | --- | --- | --- |
| *with motif filtering* |  |  |  |  |  |  |
| 5-fold cross-validation | 0.804 | 0.893 | 0.874 | 0.777 | 0.684 | 0.936 |
| Independent test | 0.786 | 0.878 | 0.861 | 0.737 | 0.698 | 0.916 |
| *without motif filtering* |  |  |  |  |  |  |
| 5-fold cross-validation | 0.657 | 0.851 | 0.814 | 0.631 | 0.588 | 0.888 |
| Independent test | 0.666 | 0.840 | 0.819 | 0.638 | 0.655 | 0.875 |

Table S8. Performance of TAPE and SeqVec on the 5-fold cross-validation and independent test^a^.

|  | Method^b, c^ | AUPRC | AUROC | Accuracy | Precision | Recall | Specificity |
| --- | --- | --- | --- | --- | --- | --- | --- |
| 5-fold cross-validation | SVM_TAPE | 0.711 | 0.862 | 0.839 | 0.703 | 0.602 | 0.917 |
|  | SVM_SeqVec | 0.726 | 0.869 | 0.846 | 0.718 | 0.618 | 0.921 |
|  | RF_TAPE | 0.683 | 0.865 | 0.824 | 0.661 | 0.588 | 0.901 |
|  | RF_SeqVec | 0.689 | 0.864 | 0.832 | 0.664 | 0.638 | 0.895 |
|  | AdaBoost_TAPE | 0.609 | 0.821 | 0.811 | 0.657 | 0.483 | 0.918 |
|  | AdaBoost_SeqVec | 0.671 | 0.850 | 0.829 | 0.706 | 0.526 | 0.928 |
|  | CNN_TAPE | 0.763 | 0.892 | 0.856 | 0.739 | 0.641 | 0.926 |
|  | CNN_SeqVec | 0.685 | 0.856 | 0.829 | 0.716 | 0.508 | 0.934 |
| Independent test | SVM_TAPE | 0.716 | 0.848 | 0.823 | 0.668 | 0.598 | 0.899 |
|  | SVM_SeqVec | 0.695 | 0.849 | 0.827 | 0.665 | 0.637 | 0.892 |
|  | RF_TAPE | 0.612 | 0.826 | 0.801 | 0.610 | 0.586 | 0.873 |
|  | RF_SeqVec | 0.605 | 0.838 | 0.817 | 0.627 | 0.675 | 0.864 |
|  | AdaBoost_TAPE | 0.615 | 0.828 | 0.781 | 0.585 | 0.454 | 0.891 |
|  | AdaBoost_SeqVec | 0.644 | 0.843 | 0.795 | 0.619 | 0.484 | 0.899 |
|  | CNN_TAPE | 0.636 | 0.827 | 0.795 | 0.616 | 0.516 | 0.889 |
|  | CNN_SeqVec | 0.563 | 0.820 | 0.781 | 0.577 | 0.473 | 0.884 |

^a^ The TAPE or SeqVec models were trained and tested on the positives-to-negatives ratios of 1:3.

^b^ Tasks Assessing Protein Embeddings (TAPE) included three modules: an LSTM, a Transformer, and a dilated residual network (ResNet) (1). We downloaded TAPE from https://github.com/songlab-cal/tape and installed it for local use. We used a small Transformer pre-trained model trained on UniRef50, which yielded a 768-dimensional feature vector for each sequence.

^c^ Sequence-to-Vector (SeqVec) was also a pre-trained protein language model, which was based on a bidirectional LSTM model with 93M parameters and was trained on UniRef50 (2). We downloaded SeqVec from https://github.com/Rostlab/SeqVec and installed it for local use. SeqVec generated a 1024-dimensional embedding vector for each sequence.


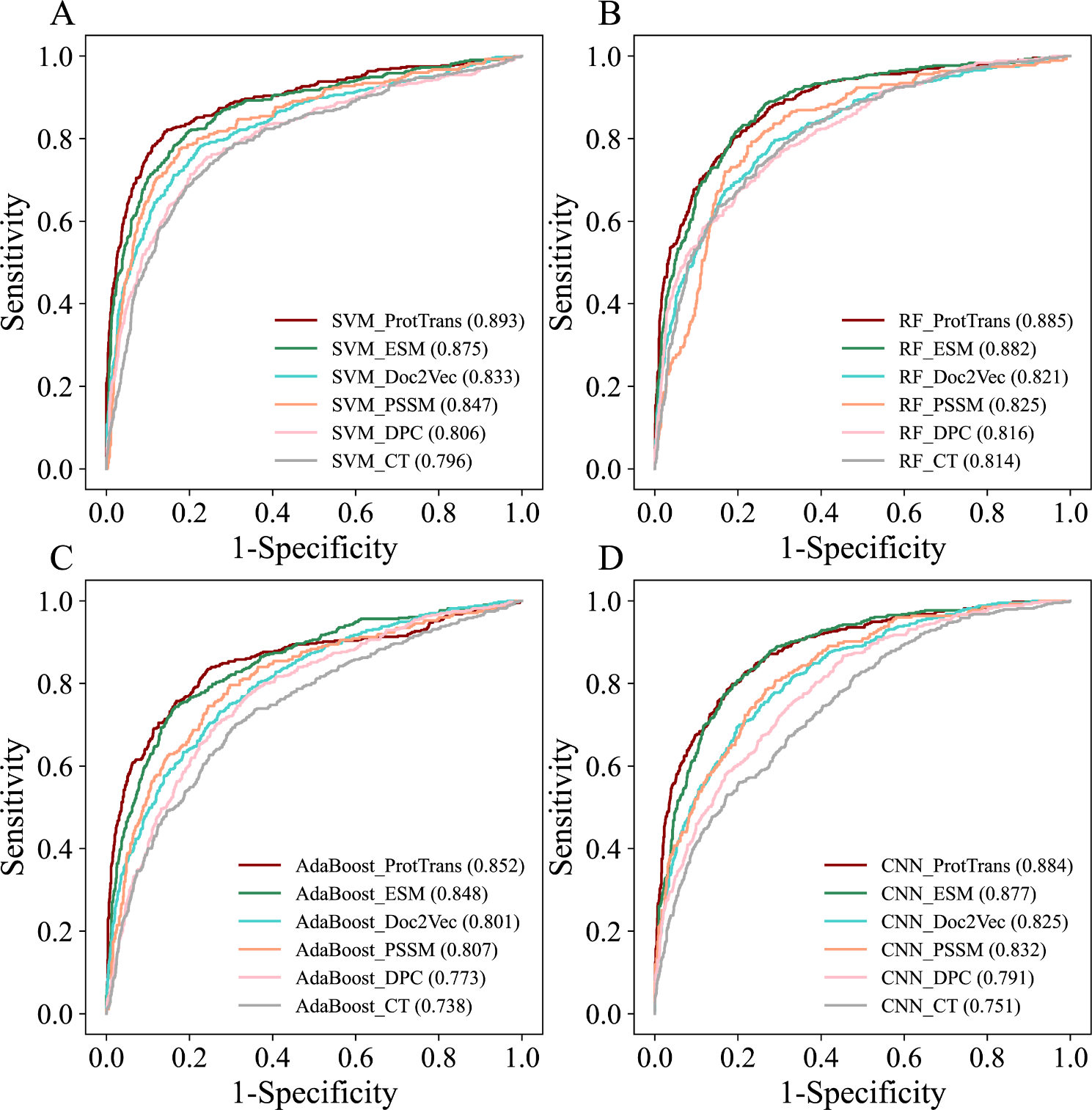


Fig. S1. Performance of various classifiers on 5-fold cross-validation. We plotted Receiver Operating Characteristics curves (ROCs) for the four machine learning models based on different sequence-based encoding schemes. Panels A, B, C and D stand for the results of SVM, RF, AdaBoost and CNN, respectively. In each panel, the parameters in brackets denote the AUROC values of the corresponding predictive models.


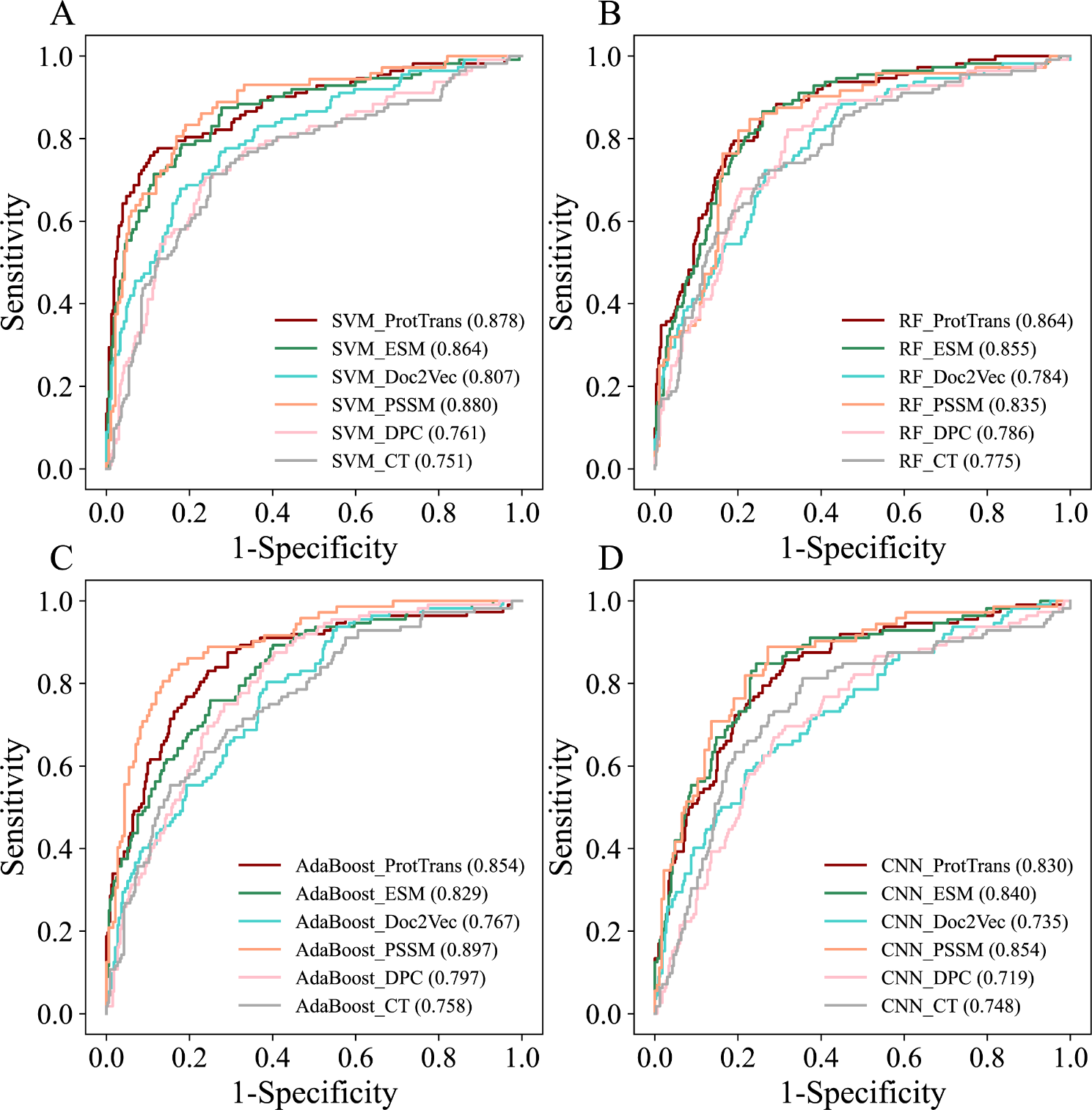


Fig. S2. Performance of various classifiers on the independent test. We plotted Receiver Operating Characteristics curves (ROCs) for the four machine learning models based on different sequence-based encoding schemes. Panels A, B, C and D stand for the results of SVM, RF, AdaBoost and CNN, respectively. In each panel, the parameters in brackets denote the AUROC values of the corresponding predictive models.


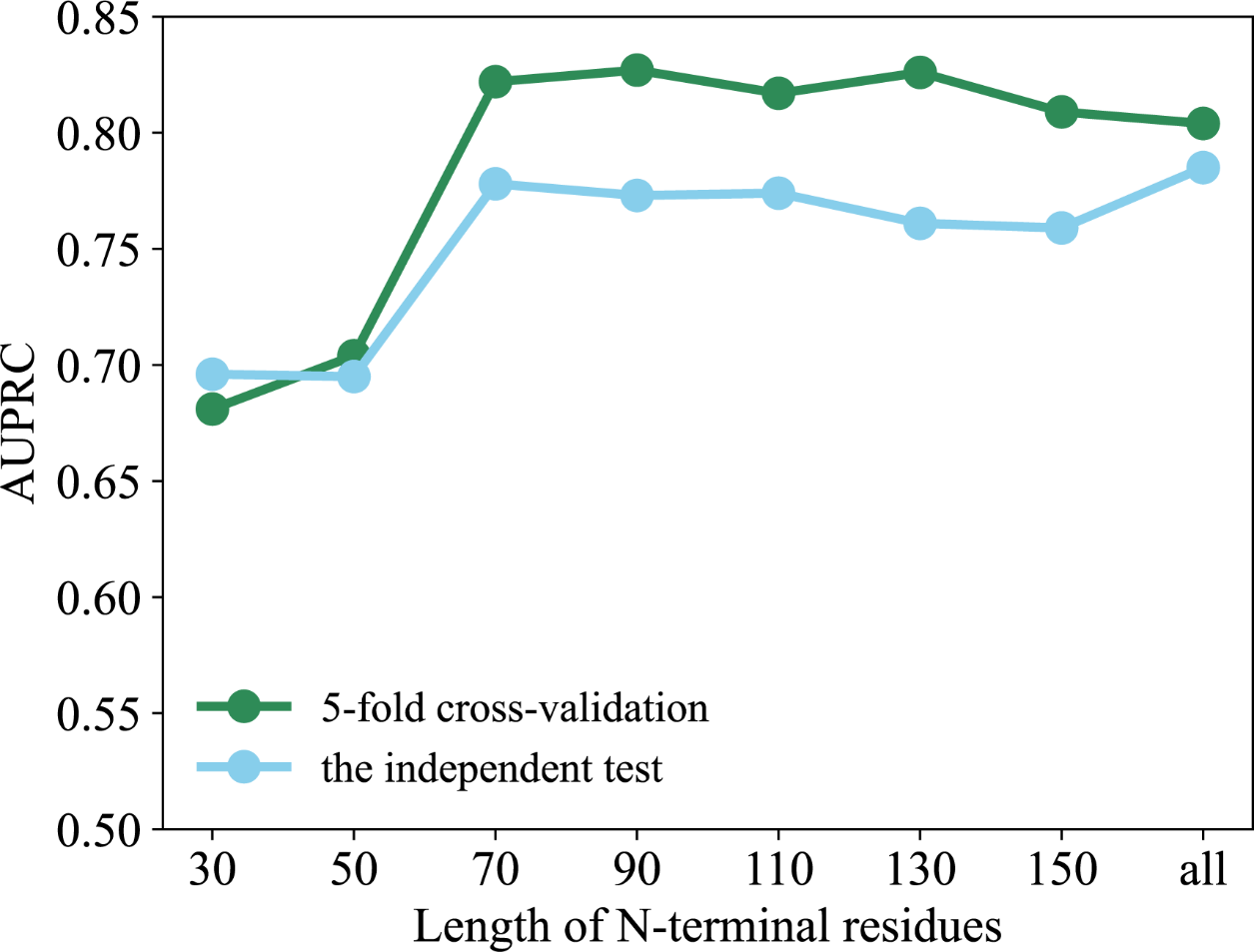


Fig. S3. AUPRCs of the SVM_ProtTrans models trained using different lengths of N-terminal residues. Note that the ratio of positives to negatives was set as 1:3 in this computational experiment.


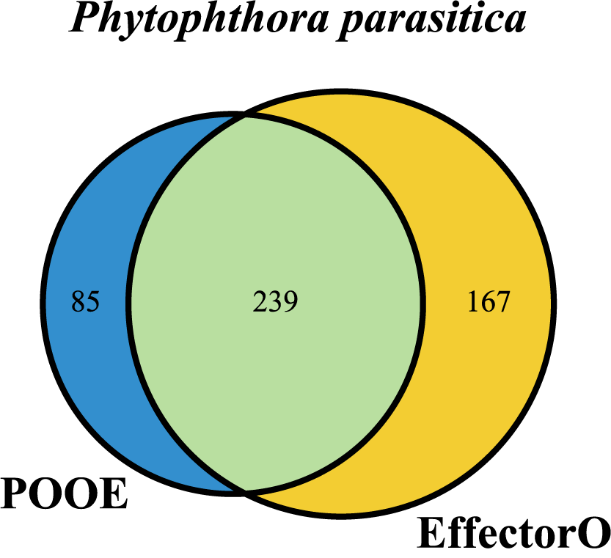


Fig. S4. Venn diagram showing the overlapping effectors predicted by POOE and EffectorO in the proteome of  *Phytophthora parasitica*. Briefly, 1515 out of the 22979 proteins in *Phytophthora parasitica* were first predicted as secreted proteins without transmembrane regions. Then, the 1515 proteins were further submitted to POOE and EffectorO to conduct proteome-wide effector identification. Since a predictive threshold at Specificity control of 89.8% [i.e., false positive rate (FPR) control at 10.2%] was provided by EffectorO, we reported both prediction results at the FPR control of 10.2% to ensure a fair comparison between POOE and EffectorO. We have made the proteome-wide prediction results of POOE and EffectorO freely available at http://zzdlab.com/pooe/index.php and https://github.com/zzdlabzm/POOE.

**References**

1. Rao R, Bhattacharya N, Thomas N, Duan Y, Chen X, Canny J, Abbeel P, Song YS. 2019. Evaluating Protein Transfer Learning with TAPE. *Adv Neural Inf Process Syst* 32:9689-9701.

2. Heinzinger M, Elnaggar A, Wang Y, Dallago C, Nechaev D, Matthes F, Rost B. 2019. Modeling aspects of the language of life through transfer-learning protein sequences. *BMC Bioinformatics* 20:723.
